# Examining the Mechanical and Thermal Properties of a Novel Hybrid Thermoplastic Rubber Composite Made with Polypropylene, Polybutadiene, S-Glass Fibre, and Flax Fibre

**DOI:** 10.3390/polym16243599

**Published:** 2024-12-23

**Authors:** Periasamy Diwahar, Karuppiah Prakalathan, K. Periyasamy Bhuvana, Krishnasamy Senthilkumar

**Affiliations:** 1 Central Institute of Petrochemicals Engineering & Technology, Chennai 600032, India; kpusha27@gmail.com; 2 Department of Mechanical Engineering, PSG Institute of Technology and Applied Research Coimbatore, Coimbatore 641062, India; kmsenthilkumar@gmail.com

**Keywords:** polymer blends, mechanical properties, thermal properties, glass fibre, flax fibre, polypropylene, polybutadiene, hybrid composites

## Abstract

In this work, twin-screw extruder and compression moulding techniques were utilized to fabricate polymer blends: polypropylene (PP), polybutadiene (PB), and composites using glass fibre (GF) and flax fibre (FF). During fabrication, the polymer ratios maintained between PP and PB were 90:10, 80:20, and 70:30. Likewise, the composites were fabricated by varying the ratios between the PP, PB, and GF, which were 90PP:10PB:10GF, 80PP:20PB:10GF, and 70PP:30PB:10GF. Additionally, a hybrid composite was fabricated by adding 20% FF to the 90PP/10PB/10GF blend. The mechanical characterization revealed that the tensile strength and modulus increased by approximately 24% and 23%, respectively, for the hybrid combination (90PP/10PB/10GF/20FF) compared to pure PP (from 21.47 MPa and 1123 MPa to 26.54 MPa and 1382 MPa). Similarly, flexural strength and impact resistance showed significant improvements in hybrid samples, with flexural strength increasing by approximately 15%. Scanning electron microscopy (SEM) was also carried out for impact-tested samples to understand the fibre-to-matrix adhesion behaviour. Regarding the DSC results, PP exhibited a melting peak between 160 °C and 170 °C. When incorporating PP into PB, a reduction in crystallinity was observed. Further, by adding GF to polymer blends, the crystallinity was increased. HDT and Vicat softening temperature results reported that the hybrid samples showed higher values of 79.3 °C and 88.2 °C, respectively, resulting in improvements of approximately 3.9% and 2.9% over standard PP. Findings from this study suggest that the novel combinations offer a promising synergy of flexibility, strength, and thermal resistance, making them suitable for medium engineering applications.

## 1. Introduction

In recent times, thermoplastic matrix composites have increasingly been used in many high-performance applications due to their excellent mechanical characteristics, lightweight nature, and manufacturability [1,2,3]. Thermoplastics’ matrices can retain their molecular structure and are also recyclable. This specific characteristic offers many environmental benefits, including recyclability, a reduced environmental footprint, lower energy consumption, waste reduction, and support for sustainable manufacturing practises with cost-effectiveness [4,5,6]. Common types of thermoplastic matrices include Polyethylene (PE), Polypropylene (PP), Polyvinyl Chloride (PVC), Polystyrene (PS), Polycarbonate (PC), Acrylonitrile Butadiene Styrene (ABS), Polyamide (Nylon), and Polybutylene (PB) [7,8].

The combination of properties such as mechanical strength, flexibility, and thermal resistance is essential for many engineering applications. However, traditional materials may not provide these combinations, particularly when lightweight and cost-effective solutions are required. Therefore, in this work, thermoplastic polymers such as PP and PB were selected and blended due to their inherent properties. PP provides durability, processability, and mechanical properties, while PB improves flexibility and impact resistance. Thus, fabricating this blend can be suitable for various applications. To further enhance the samples’ properties, GF was added to improve their strength and thermal behaviour, while FF was added to reduce environmental impact and enhance recyclability. These combinations provide good performance due to the addition of multiple fibres, such as GF and FF. However, GF introduces challenges for recycling and reusability, which this experimental study acknowledges as a critical area for future work.

Polypropylene (PP) is a significant polymer in the field of commodity plastics. It is widely used in various industries and as a material for everyday products due to its high crystallinity, low density, chemical resistance, and convenient manufacturing process. However, its limited impact toughness and low melt strength greatly restrict its potential for broader applications [9,10]. Researchers are exploring blending with different polymers to expand its application possibilities. Studies have found that blending different types of elastomers with polypropylene improves not only its impact strength but also its melt flow behaviour, influencing its ease of processing and crystallization [11]. On the other hand, the research on PP/elastomer composites has primarily been limited to the use of the following elastomers: the styrene–ethylene–butylene–styrene triblock copolymer (SEBS) [12], the copolymer of the ethylene–propylene–diene monomer (EPDM) [13], and ethylene–propylene rubber [14,15]. When investigating blends with PP, it is rare to select PB, a less expensive and simpler elastomer. PP, a glass fibre–rubber composite material, has excellent strength, resistance to corrosion, and flame-retardant qualities. It is prepared using an efficient and cost-effective approach [16,17]. The usage of synthetic fibres with various thermoplastic matrices has been studied to assess their suitability through mechanical, physical, morphological, and thermal characterization techniques. For example, Sadr Kenari et al. [18] investigated the mechanical behaviour of PP/GF-reinforced composites. The results reported that adding a 40% weight fraction of GF to PP composites increased their tensile strength by 91% and flexural strength by 62%. In another work, Raghvan et al. studied the mechanical characteristics of PP/GF/ethylene–propylene–diene–rubber–(EPDM) ternary composites. The results showed that the impact strength of PP/GF15/EPDM20 was increased by 56% compared to pure PP [19]. The incorporation of natural fibres as reinforcing materials in PP matrices has been an increasingly significant field of study. Scientists have dedicated significant resources to the creation and assessment of polymer composites that are strengthened by different types of plant fibres [20,21]. Some examples of plant fibres are flax [22,23], jute [24], hemp [25], pineapple fibres [26], sisal fibres [27], and banana fibres [28], which are obtained from the agricultural waste of the relevant economically important products. Flax fibres, like lignocellulosic biomass, are predominantly composed of cellulose, hemicellulose, and lignin [29,30]. To improve the performance of composites, researchers have fabricated hybrid composites using two fibres with a single matrix. They have also developed polymer-blended samples and examined their performance. For example, Durvasulu et al. [31] fabricated hybrid composites using flax/ramie/phenol formaldehyde composites and examined their mechanical and thermal properties. The results reported that the hybrid samples exhibited improved thermal stability, evidenced by temperature regions ranging between 270 °C and 630 °C. This enhancement was attributed to the enhanced interaction between the fibres and matrix. In another work, researchers [32] investigated the reinforcement effect of grape fibre with different matrices such as PS, high-density polyethylene, and polyoxymethylene and subjected them to mechanical and thermal studies. The researchers reported that the flexural strengths increased with the addition of fibre loading. For instance, 30 wt.% of fibre-loaded samples exhibited a twofold improvement compared to pure high-density polyethylene. The TGA and differential thermal analysis results showed that burning was delayed since the fibres burned before the thermoplastic matrices.

In another work, Cho et al. [33] examined the performance of thermoplastic composites made by polyketone and carbon fibre, whereby the carbon fibre loading was varied from 0% to 30%. The results reported that the mechanical behaviour, thermal stability, and conductivity of the composites improved with the incorporation of carbon fibre into the polyketone matrices. Anjum et al. [34] fabricated polymer-blended samples using polyetheretherketone (PEEK)/polyethersulfone (PES)/carbon fibre and examined their performance in terms of mechanical and thermal properties. The results reported that mechanical properties were improved by adding carbon fibres. Moreover, the carbon fibres increased the crystallinity of the PEEK matrix, and thus enhanced the thermal conductivity and thermal stability of the CF/PEEK composites.

This characteristic of glass and flax fibres is responsible for the superior mechanical properties of PP, GF, and FF composites, as well as their strong resistance to moisture and chemicals. The role of FF is not limited to environmental impact; it will hybridize with synthetic fibre and develop on lightweight composites. GF enhances the mechanical and thermal insulation properties of composite materials, making them more suitable for their intended applications. Recycling FF contributes to the growth of the circular economy [35,36,37].

In this work, the authors fabricated PP/PB blends using melt blending techniques and composite laminates were prepared by compression moulding. To the authors’ knowledge, this is the first experimental work to develop polymer blends using a combination of PP and PB in ratios of 90:10, 80:20, and 70:30. Similarly, the composites were developed by altering the proportions of PP, PB, GF, and FF to 90PP:10PB:10GF, 80PP:20PB:10GF, 70PP:30PB:10GF, and 90PP:10PB:20FF:10GF. Additionally, G and FF were reinforced with the polymer blends to improve mechanical (tensile, flexural, and impact properties) and thermal properties (DSC, HDT, and Vicat softening temperature). The morphology of both polymer blends and composites was thoroughly examined using scanning electron microscopy (SEM).

The motivation behind this experimental work was to examine the synergistic effects of combining PP and PB matrices with GF and FF to develop hybrid composites with superior mechanical and thermal characteristics. Furthermore, specific ratios between polymers and reinforcements were selected to optimize parameters such as flexibility, strength, and thermal stability. The fabricated hybrid samples could be suitable for medium engineering applications using these combinations.

## 2. Materials and Methods

### 2.1. Materials

A polypropylene (PP) impact copolymer, polybutadiene (PB), S-glass fibre (GF), and flax fibre (FF) were used to fabricate the composite samples. The PP MI3030 was purchased from Reliance Industries Limited in Mumbai, India. The PB rubber granules were purchased from Enterprising Polymer, Chennai, India. GF and FF were purchased from Go-green Goods, Chennai, India. The essential properties of the polymers and fibres are given in Table 1 and were collected from the relevant material data sheets.

The fibres were provided in the form of finished fabrics rather than original fibres. These finished fabrics ensure uniformity in fibre dimensions and help improve compatibility with polymers during fabrication. Finished fabrics provide consistent reinforcement and are easier to handle during the fabrication process, especially in hot press moulding. These properties contribute to the enhanced mechanical and thermal performance of the fabricated samples.

### 2.2. Composite Fabrication

Twin-screw extruder and compression moulding machines were used to fabricate the polymer blends and the composite samples. A twin-screw extruder machine was chosen due to its superior mixing capability, which allows for the uniform dispersion of consistent PP and PB, and compression moulding was used to increase composite density and structural stability. This process consolidates extruded materials with regulated heat and pressure, improving interlayer bonding. Twin-screw extruder and compression moulding were chosen to improve composite mechanical and thermal properties.

**Figure 1:**
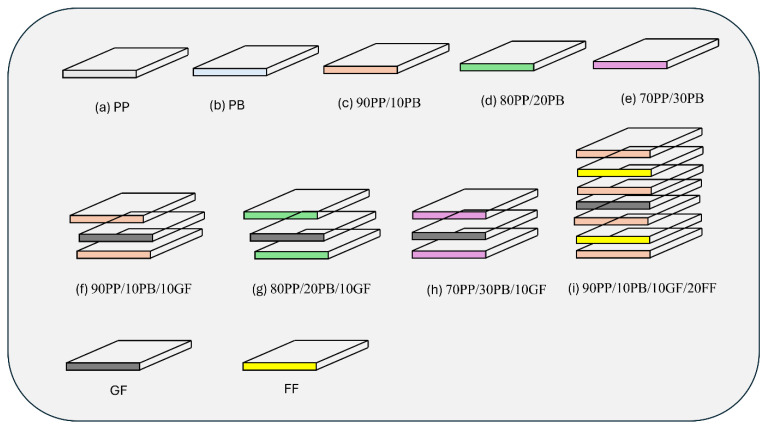
Stacking sequence details of polymer blends and their composites.

Initially, the PP and PB were dried for 2 h at 60 °C. Then, using a high-speed mixer, the polymers were utilized in their original forms (such as 100:0 for pure PP, and 0:100 for pure PB) or mixed in different ratios: 90:10, 80:20, and 70:30. The various compositions of PP, PB, GF, and FF are given in Table 2. The polymer mixtures were produced using twin-screw extruders in temperature ranges from 190 °C to 230 °C. The extrusion speed was maintained at 400rpm. The resultant materials from the twin-screw extruder were further utilized to make sheets through a compression moulding machine. The extruded materials were carefully placed in the heated platens of the compression moulding machine. The compression moulding machine gradually increased the moulding temperature to 210 °C, and pressure was maintained, ranging from 40 to 50 bars. These conditions were maintained for 10 min, which allowed the materials to cure and the different components to be fused into homogeneous composite samples. Figure 1 shows the various stacking sequences of polymer blends and their composites. The fabricated samples were allowed to cool down to 40 °C from 70 °C with pressure to prevent the formation of internal stresses. After reaching the cool temperature, the pressure was relieved. Then, the newly formed samples were removed from the mould, cut per ASTM standards, and subjected to testing.

### 2.3. Characterization

#### 2.3.1. Tensile Test

A tensile test was conducted per the ASTM D638 [38] using a universal testing machine equipped with a 100 kN load cell. The test was conducted at a room temperature of around 25 °C. A cross-head and a gauge length of 2 mm/min and 50 mm were maintained. Five identical samples were used, using a sample length of 165 × 25 × 3 mm. Tensile strength is a material’s tension-breaking resistance. High tensile strength protects helmets from hits and stress by resisting pulling.

#### 2.3.2. Flexural Test

The flexural test was conducted per the ASTM D790 [39] using the universal testing machine equipped with a 100 kN load cell using a 5 kN load cell. The test was performed at room temperature, around 25 °C. The loading rate was maintained at 2 mm/min. Five identical samples were used using 127 × 12 × 3 mm dimensions, ensuring the repeatability of results. Helmets must endure bending and flexing, especially during impacts involving various angles of force. Flexural strength allows the helmet to absorb and redistribute these forces, minimizing breakage.

#### 2.3.3. Impact Test

An Izod impact test was conducted per the ASTM D256 [40] using a Tinius Olsen machine (Tinius Olsen Testing Machine Co., Horsham, PA, USA). The impact tester was equipped with a 4.537 kg hammer. Sample dimensions of 65 × 13 × 3 mm were used, and the repeatability of impact results was ensured using five identical samples. A material’s impact strength reveals its ability to absorb energy and resist impact without breaking. It is essential for helmet shock absorption testing.

#### 2.3.4. Scanning Electron Microscopy (SEM)

Fractured samples were subjected to an SEM test using a Hitachi S4700 machine (Hitachi High-Tech Corporation, Tokyo, Japan). SEM was used to understand the behaviour of fibre-to-matrix bonding. Before testing, all the fractured samples were gold-coated. Gold coating is generally used to improve the electrical conductivity of a sample surface with a beam of electrons. In addition, the conductive surface allows for obtaining good imaging and analysis results.

#### 2.3.5. Differential Scanning Calorimetry (DSC) Test

A DSC test was conducted according to ASTM D3418 [41] using a PerkinElmer machine (PerkinElmer, Inc., Waltham, MA, USA) under a nitrogen atmosphere. The heating rate was adjusted to 10 °C, while the scanning speeds of the machine ranged from 0.01 to 100 °C, encompassing a temperature range of −70 to 200 °C. Various aspects of the environment can affect helmets. The stability of the helmet material and its protection against degradation or loss of protective characteristics due to temperature changes can be ensured by understanding its thermal behaviour.

#### 2.3.6. Heat Deflection Temperature (HDT) and VICAT Softening Temperature

The HDT and VICAT softening temperature tests were conducted per the ASTM D648 [42] and ASTM D1525 [43], respectively, using an HDT/VICAT instrument (ZwickRoell GmbH & Co. KG, Ulm, Germany). During the test, the samples were subjected to a constant load of 0.45 MPa and were positioned to induce bending stress due to an increase in temperature. The heating process was accomplished using a silicone oil bath at 120 °C/h. The VICAT softening temperature was determined using technique B. The samples were exposed to a steady tension of 50 N while heated at 50 °C/h. Helmets may be susceptible to high temperatures, either from the environment or from frictional heat during an accident. HDT and VST determine the temperature at which a material deforms under a specific load. They indicate the material’s capacity to endure heat without losing its mechanical characteristics.

## 3. Results and Discussions

Determining the mechanical and thermal properties of the composites, as well as understanding the chemical and structural features of the polymer blends, are highly important. PP is well known for its semi-crystalline nature, including a highly ordered molecular structure. This contributes to its strength, thermal properties, and stiffness. Despite these advantages, PP has lower impact toughness, which could limit its broader applications.

Regarding PB, it is an amorphous elastomer with a flexible molecular backbone. This flexibility can disrupt the crystallinity of PP polymers when added, resulting in improved flexibility and impact resistance. The inclusion of fibres like GF enhances stiffness and strength due to their higher modulus. On the other hand, FF contributes to ductility and toughness because of its fibrous nature. Therefore, the use of these polymers and fibres can significantly influence overall material performance.

### 3.1. Tensile Strength

**Figure 2:**
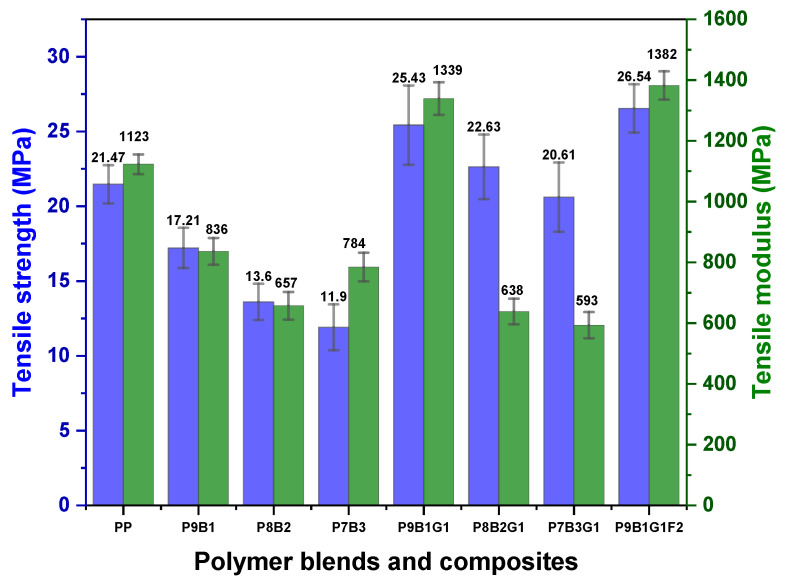
Tensile strength and modulus of PP, PB, polymer blends, and their composites.

**Figure 3:**
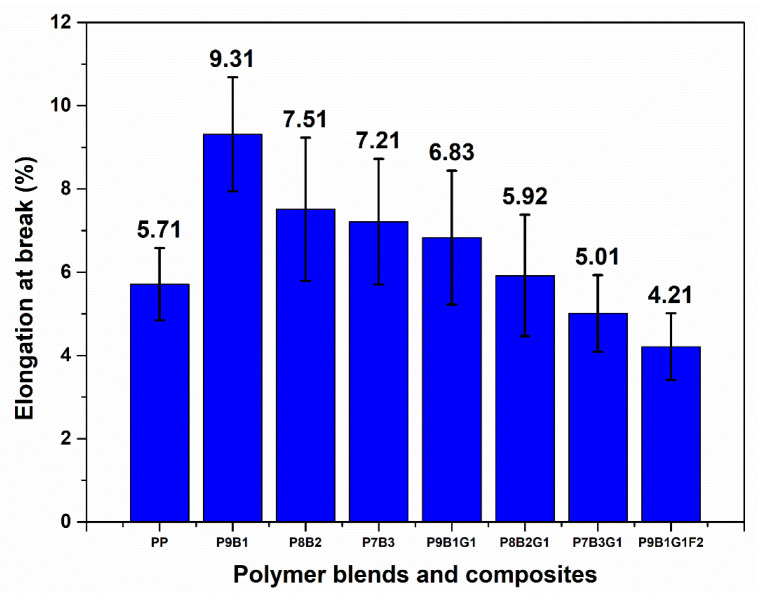
Elongation at break of PP, PB, polymer blends, and their composites.

Figure 2 and Figure 3 illustrate the tensile properties of the pure polymers, polymer blends, and their composites.

The pure polymers show distinct results that reflect the behaviour of their molecular structures. PP samples exhibited higher tensile strength (~22 MPa) than pure PB (4.28 MPa ± 0.51). PP is a semi-crystalline polymer that exhibits a tightly packed molecular arrangement and strong intermolecular forces that are responsible for its high tensile strength and rigidity [44]. This characteristic is attributed to the higher degree of crystallinity in PP polymers, where the tightly packed chains limit segmental movements. This enables the material to withstand higher pulling forces with less deformation, making it suitable for structural applications [9]. In addition, PP exhibited a high tensile modulus value (1123 MPa), indicating its stiffness and resistance against deformation under tensile stress. Conversely, PB exhibited lower tensile strength (4.28 MPa) and a lower tensile modulus (0.47 MPa). This was ascribed to its amorphous nature and weaker intermolecular forces [45]. These values reflect PB’s inherent flexibility, ductility, and ability to undergo extensive elongation (907.33% ± 97.08) before failure. However, the addition of polybutadiene (PB) improves the blend’s molecular flexibility due to PB’s amorphous structure and low intermolecular forces. The presence of PB disrupts the crystalline regions in PP, which leads to a reduction in crystallinity and a decrease in the overall tensile strength and modulus because of this flexibility. The tensile strength of polymer blends (P9B1, P8B2, and P7B3) decreased with increasing PB and ranged from ~12 MPa to ~18 MPa. This trend was aligned with the pure PB’s inherent strength relative to PP. Among the samples, P9B1 exhibited a favourable balance between PP’s strength and PB’s flexibility. Like tensile strength behaviour, the tensile modulus of the polymer-blended samples (P9B1, P8B2, and P7B3) decreased with increasing PB loading, ranging from ~658 MPa to ~837 MPa. This decrease was attributed to the reduced stiffness of PB samples compared to PP samples, which allowed the blends to exhibit greater compliance under tensile loading conditions [46].

Regarding elongation at break, the polymer blends (P9B1, P8B2, and P7B3) exhibited values ranging from 7.21% to 9.31%, as shown in Figure 3. These ranges indicate moderate to higher ductility values in all the samples. In addition, PB’s inherent ductile nature allowed the samples to undergo significant deformation before failure, such as 7.21% to 9.31% [47]. Among the samples, P9B1 exhibited the highest elongation at break values (7.51%) due to a higher amount of PB, which enhanced the polymer blend’s ability to stretch without failure.

The decrease in modulus and elongation was attributed to the disruption of the PP polymer’s crystalline structure caused by the addition of PB polymers. Since PB introduces amorphous regions, the modulus of the samples decreased, weakening their ability to resist deformation. Additionally, the introduction of GF and FF improved stiffness; however, these fibres restricted the elongation of the samples due to their brittle nature. Consequently, the combined factors led to a reduction in the modulus and elongation of the composite samples.

Furthermore, the tensile strength of polymer blends was improved by adding GF. For example, the tensile strength increased from 17.21 MPa in 90PP/10PB to 25.43 MPa in P9B1G1, representing a 47% enhancement with 10% GF. Additionally, increasing the PB content from 10 to 30% resulted in a decrease in tensile strength. However, in GF-reinforced combinations, PB contributed to the overall strength of the composite due to its higher elongation at break and energy absorption capabilities before failure. Despite the decrease in tensile strength with the addition of PB content, GF-reinforced samples exhibited higher tensile strength values ranging from 11.9 MPa to 17.21 MPa in the polymer-blended samples, while the GF-reinforced samples ranged from 20.61 MPa to 25.43 MPa.

The mechanical performance of the composites was further enhanced by adding FF to P9B1G1, as shown in Figure 3. The composite exhibited a tensile strength of 26.54 MPa due to the synergistic combination effects of GF and FF. Both fibres resulted in additional strength as well as load-carrying capacity. Anni Wang et al. reported that the combination of GF and FF generates a hybrid effect in which the drawbacks of one type of fibre are balanced by the strengths of the other. GF provides high stiffness and strength, while FF improves toughness and reduces the density of the composite, resulting in a well-balanced material with excellent mechanical performance [48], resulting in improved tensile strength and modulus values.

Regarding elongation at break, the P9B1G1F2 hybrid combination reduced ductility compared to pure PP and pure PB samples. This was attributed to the incorporation of GF and FF. These fibres limited the composite’s ability to deform before breakage. Nevertheless, the P9B1G1F2 hybrid sample resulted in less ductility and may have been more brittle. This behaviour was compensated by the improved tensile strengths resulting from adding GF and FF. In addition, the P9B1G1F2 sample exhibited a high tensile modulus value (1382 MPa). This improved modulus value indicates that the hybrid sample effectively distributed loads and stresses.

### 3.2. Flexural Strength

**Figure 4:**
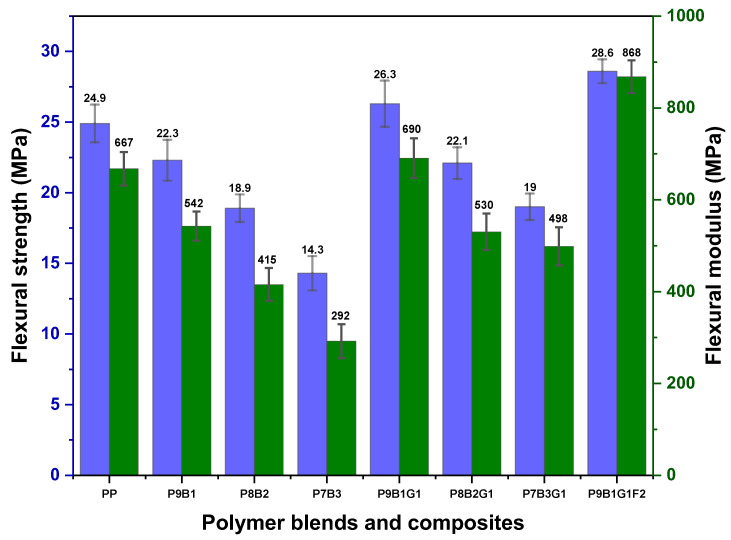
Flexural strength and modulus of PP, PB, polymer blends, and their composites.

Figure 4 shows the flexural strength and flexural modulus of the pure PP, polymer blends, and their composites.

Pure PP exhibits a flexural strength and modulus of ~25 MPa and ~670 MPa. PP is a semi-crystalline thermoplastic with higher stiffness and strength due to its regular molecular structure. Thus, PP has a higher flexural strength and modulus because its crystalline regions can bear and distribute loads effectively [49]. On the other hand, PB is a synthetic rubber known for its higher elasticity and absorption impact without fracturing. Moreover, PB has an amorphous structure with lower crystallinity values [50]. This makes the PB samples resist less deformation under loading conditions. Therefore, the flexural properties of the polymer-blended samples (P9B1, P8B2, and P7B3) decreased when the PB content was increased from 10% to 30%. This reduction in flexural properties indicates that adding PB to PP makes the samples less stiff and less strong in flexure properties.

The incorporation of GF improved the flexural properties of the polymer blends, compensating for the loss of stiffness and strength caused by the PB. GF is a high-strength and high-modulus fibre, which helped improve the flexural strength and modulus properties [51]. For instance, the P9B1G1 combination exhibited higher flexural strength (26.3 MPa) and flexural modulus (690 MPa) than pure PP. This improvement could be due to the reinforcing effect of GF, which compensated for the softening effect provided by PB. In this case, GF provided effective reinforcement and distributed the loads, adding rigidity by overcoming the potential decrease in flexural properties due to the addition of PB. When increasing the PB loading to 20% in the P8B2G1 composite, the flexural properties decreased compared to the P9B1G1 composite because the increased loading of PB increased the flexibility and lowered the stiffness of the composite, whereby GF could not fully compensate. Moreover, the reduction in PP polymer loading reduced the overall composite crystallinity and stiffness since the load-carrying ability of PP polymers was diluted due to the increase in PB [52]. When further increasing the loading of PB in the P7B3G1 composite, the flexural properties decreased, which was consistent with the observation that the addition of PB led to lowered stiffness and strength. Nevertheless, GF provided reinforcement, and the increased order of PB reduced the composites’ ability to resist deformation under flexural loading conditions.

Increasing the PB loading to the composite samples (P9B1G1, P8B2G1, and P7B3G1) from 10% to 30% introduced more amorphous regions and flexibility to the composites. Moreover, PB acted as a plasticizer within the PP polymers, reducing overall stiffness and strength values. Due to the addition of GF, this effect was somewhat reduced in the polymer-blended composites, and flexural properties were enhanced compared to pure polymers and polymer-blended samples. Therefore, GF improved the composites’ bending rigidity by offering structural reinforcement, while PB provided flexibility by behaving as a plasticizer. This harmonious combination of properties was achieved in these hybrid materials. This equilibrium allowed the composites to attain moderate flexibility and enhanced stiffness, thereby expanding their potential for use in structural components that require a certain degree of flexibility. The flexural properties of the composite samples were further enhanced by introducing FF, a natural fibre. The FF helped enhance the flexural performance and contributed to environmental sustainability. Though the FF has a lower modulus when compared to GF, the introduction of FF enhanced the strength and modulus values owing to its fibrous structure and good bonding nature with polymers. Yongli Zhang et al. reported that a hybrid composite structure enhances stress distribution and improves resistance to crack development. Glass fibres (GFs) serve as obstacles to the initiation of cracks, while FF contributes to connecting and stopping the propagation of cracks, hence preserving the structural integrity of composites and improving their tensile properties [53]. GF and FF contributed a synergistic effect in the P9B1G1F2 composite, providing dual reinforcement within the PP and PB polymers. GF provided higher stiffness and strength, while FF offered additional reinforcement and increased the overall performance of the composite sample. FF could help transfer stresses effectively across the composite due to its fibrous nature and interaction with GF. Although PB reduced the stiffness of the composite owing to its elastomeric nature, the synergistic effects of GF and FF compensated for these softening effects. Thus, the high modulus of GF and the reinforcing ability of FF jointly ensured that the P9B1G1F2 composite remained stiff and strong despite the addition of PB.

### 3.3. Impact Strength

**Figure 5:**
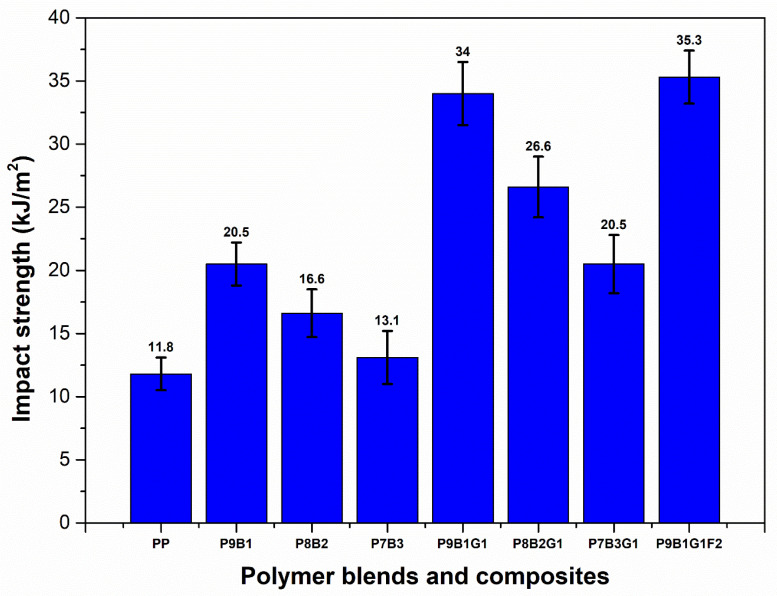
Impact strength of PP, PB, polymer blends, and their composites.

Figure 5 shows the impact strength of PP, polymer blends, and their composites. PP exhibited a moderate impact strength of ~12 kJ/m^2^ as a semi-crystalline thermoplastic polymer. The PP’s structure allowed for limited energy absorption before its failure; thus, it is not suitable for high-impact applications. Moreover, the impact value of ~12 kJ/m^2^ suggests that the PP has moderate toughness. Introducing the PB polymer with PP in the P9B1 sample significantly improved the impact strength to ~21 kJ/m^2^. This enhancement indicates that PB’s elastomeric characteristic helped absorb energy in the composite sample [54]. Moreover, PB is a rubbery material that can deform under impact loading without fracture, thus dissipating more energy effectively than rigid PP polymers. P9B1 exhibited the highest impact strength of ~21 kJ/m^2^ among the polymer-blended samples. In addition, this combination can balance PP’s stiffness and PB’s elasticity for improved energy absorption. When increasing the PB loading to 20% and 30%, the impact strength decreased in the P8B2 and P7B3 samples. Though PB polymers contributed to toughness characteristics, the excessive addition of PB reduced the load-bearing capacity. This reduction was attributed to the fact that excessive loading of PB could prevent effective stress transfer and the dissipation of impact energy across the polymer-blended samples. In addition, the PB’s over-softening effect could reduce the energy transfer and dissipation mechanisms.

The impact strength of polymer-blended samples was significantly improved to 34 kJ/m^2^ by introducing GF in the P9B1G1 composite sample. GF’s higher tensile strength and stiffness helped distribute the impact energy effectively in the composite samples and thus prevented failure and improved their overall toughness property [55]. However, when increasing the PB loading in P8B2G1 and P7B3G1, the impact strengths were reduced to ~27 kJ/m^2^ and ~21 kJ/m^2^, respectively. These results suggested that the increasing flexibility and lower stiffness from the addition of the PB polymer significantly influenced the composite’s capacity to absorb and dissipate impact energy. However, the impact strength of the composite samples was higher than that of the polymer-blended samples.

The impact strength of composite samples was further enhanced by adding FF to the P9B1G1 sample. It was observed that among all samples tested, the P9B1G1sample exhibited the highest impact strength due to the combined effect of GF and FF. Since FF is a natural fibre, it can introduce additional mechanisms in energy absorption due to its microstructure [56]. In addition, FF’s fibrous nature could help enhance the interfacial adhesion characteristics between the fibres and polymers, improving the load transfer and the samples’ toughness behaviour.

### 3.4. Scanning Electron Microscopy (SEM)

**Figure 6:**
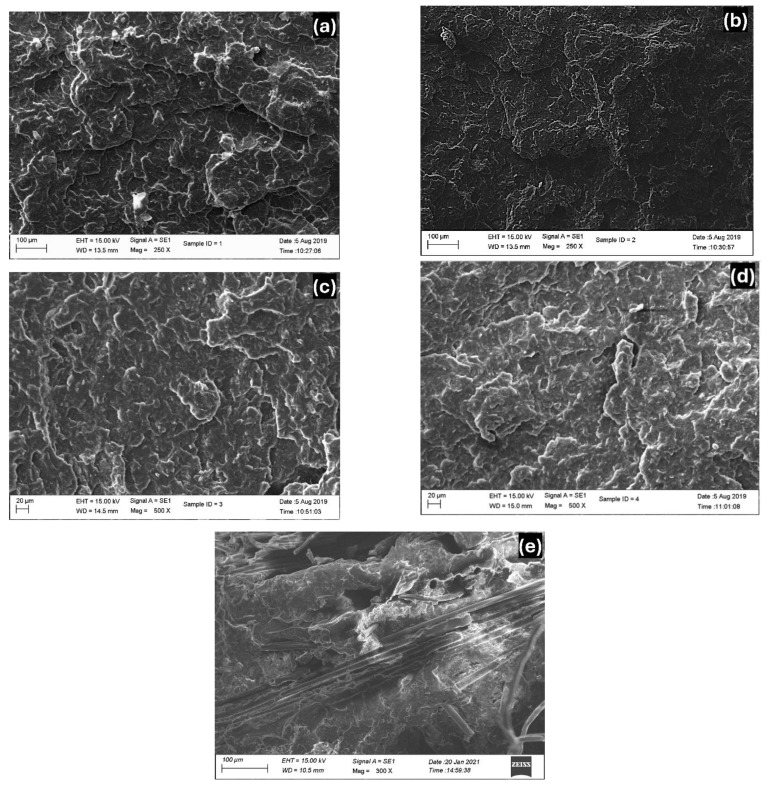
SEM images of impact tested samples: (**a**) PP, (**b**) P9B1, (**c**) P8B2, (**d**) P7B3, and (**e**) P9B1G1F2.

The morphology of the samples was analyzed using SEM images (Figure 6) of impact tested samples, which confirmed their described structure. For instance, the images provide evidence of fibre dispersion, the homogeneity of the polymer blends, and fibre-to-matrix bonding. These features help explain the described structure and provide details about material interactions under mechanical loading conditions.

A highly textured fracture surface of the PP sample is shown in Figure 6a. This observation can be correlated with the significant plastic deformation of the sample and suggests that the pure PP sample absorbed a moderate amount of energy before failure. Figure 6b displays a rougher surface and indicates that the P9B1 sample could absorb more impact energy, reflecting an improved impact strength value (20.5 kJ/m^2^). Debonding at the interfaces was noticed in some areas in Figure 6c,d. Thus, the poor bonding may have reduced the impact strength of the samples. Figure 6e shows that the fibres are bonded with the polymer matrices and not detached from the matrices. This observation indicates good interfacial adhesion, which requires higher energy to break the samples and thus contributes to higher impact strength, as reported in Figure 5.

### 3.5. Differential Scanning Calorimeter (DSC)

**Figure 7:**
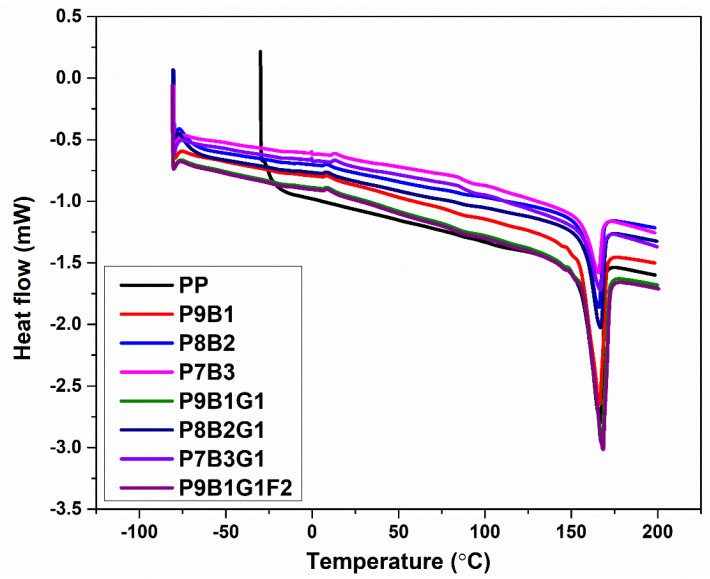
DSC plot of PP, PB, polymer blends, and their composites.

Figure 7 displays the DSC plot for the PP, polymer blends, and composites. The PP demonstrates its typical semi-crystalline behaviour with a melting transition. Moreover, its high stiffness and strength can be evidenced by the sharp melting peak around 160 °C to 170 °C, indicating the good crystallinity of PP. As for the polymer blends, the P9B1 sample exhibited a broader and lower temperature curve than PP. This was attributed to a reduction in crystallinity. Because the PB is amorphous [9], the crystalline structure of PP was disrupted by the addition of PB in the P9B1 sample. This reduction in crystallinity can be correlated with the improved impact strength [57] of the P9B1 sample (refer to Figure 5) because PB can absorb more energy and dissipate energy compared to the rigid PP.

With an increase in PB loading to 20% in the P8B2 sample, the crystallinity of PP was further disrupted (refer to Figure 7), and the curve broadened with a slight decrease in the melting temperature. This reduction can be correlated with a reduced flexural property, as seen in Figure 4. Further increasing the PB loading to 30% in the P7B3 sample resulted in the lowest and broadest melting peak (Figure 7). Among the blended samples, P7B3 had the lowest degree of crystallinity, which could be associated with its lower flexural property.

GF was added to reinforce the polymer blends, and their DSC behaviour was examined. Figure 7 illustrates that the melting behaviour of the P9B1G1 composite displayed a slight decrease in crystallinity compared to the pure PP. This was attributed to the presence of the PB and GF. The melting peak appeared around 155 °C and ended at 175 °C. When PB was increased to 20% in the P8B2G1 composite sample, the peak broadened and shifted to a lower temperature. These observations could be due to the influence of amorphous PB and its plasticizing effect. With a further increase in PB loading to 30% (P7B3G1), the crystallinity of the sample was greatly reduced. This observation was aligned with a decrease in the sample’s mechanical properties, such as stiffness and strength.

FF was introduced in the P9B1G1F2 composite sample, and it was noticed that the melting temperature slightly increased compared to the samples blended with PB and GF. Based on the onset of melting temperatures, FF appeared to enhance the crystalline structure of PP. This could be beneficial in increasing impact strength due to the energy absorption capacity of FF without compromising stiffness and strength values.

Based on the observed DSC results, the stability of the hybrid composite samples under varying thermal conditions is highlighted. The incorporation of GF and FF slightly enhanced the crystallinity of PP, leading to improved thermal resistance. This behaviour is particularly significant for various applications, especially in automotive components, where materials are exposed to elevated-temperature environments during operation.

### 3.6. Heat Deflection Temperature (HDT)

**Figure 8:**
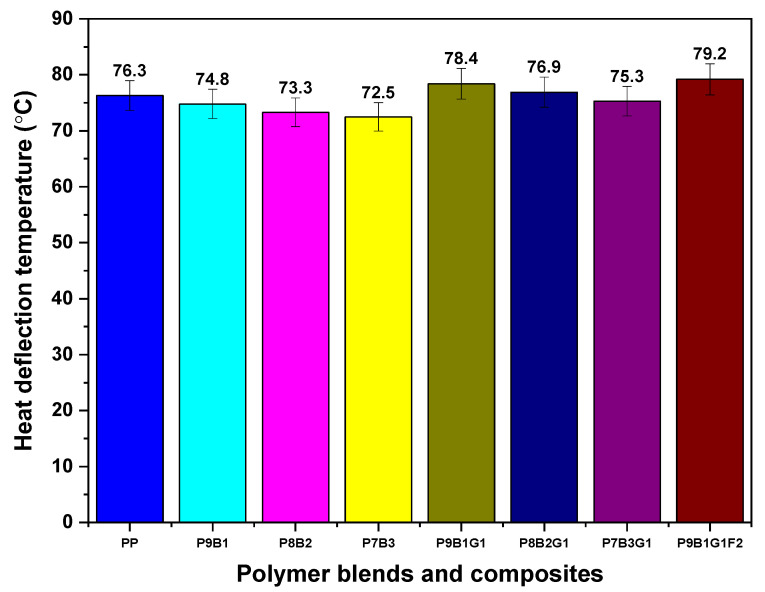
HDT of PP, PB, polymer blends, and their composites.

Figure 8 presents the HDT of the PP, polymer blends, and their composites. The HDT test is helpful in predicting a material’s reliability and integrity in applications where mechanical stress and heat are factors [58]. The temperature at which the material deforms under specific loading conditions is measured during the test, indicating the material’s behaviour in heated conditions while bearing weight.

The PP sample had an HDT value of 76.3 °C, setting the baseline for the polymer blends and their composites. As a semi-crystalline polymer, the PP exhibited a moderate HDT value, as shown in Figure 8 [59]. When incorporating 10% PB into the P9B1 sample, the HDT value decreased to 74.8 °C. Due to PB’s amorphous nature, it softened the polymer blend, causing it to deform under load as the temperature increased.

When PB loading was increased to 20% in the P8B2 sample, the HDT was further reduced to 73.3 °C. This decrement in HDT was ascribed to the amorphous nature of the PB polymer. The higher proportion of PB thereby reduces stiffness at higher temperatures. A similar trend continued for the P7B3 sample, whereby PB was loaded for 30%.

In addition, the HDT of GF-reinforced samples (P9B1G1, P8B2G1, and P9B1G1F2) improved when compared to polymer-blended samples. This is because of GF’s high strength and modulus, which contributed to improving the stiffness and thermal stability of the samples [60]. However, with the increase in PB loading in P8B2G1 and P9B1G1F2 composites, there was a slight decrease in HDT, as observed in Figure 8. These observations indicate that GF helped in enhancing the HDT of composite samples, but increasing PB’s loading influenced the overall thermal stability of composite samples.

By introducing FF with composite samples to the P9B1G1F2 sample, the HDT was further improved to 79.2 °C. This value was the highest among the tested polymers, polymer blends, and composites. Thus, the FF improved thermal stability due to its inherent thermal resistance property and good interaction ability [61].

Based on the observed results from Figure 8, the HDT values were improved with the addition of fibres, making the samples superior to pure PP for thermal-based applications. Moreover, the hybrid samples could be utilized in high-temperature applications. For instance, the P9B1G1F2 sample exhibited an HDT value of 79.2 °C, surpassing the threshold value of PP. These improved values suggest that the hybrid samples are suitable for applications such as under-hood automotive parts and medium-load components.

### 3.7. Vicat Softening Temperature (VST)

The Vicat softening temperatures (VSTs) give the composite materials thermal stability and suggest potential applications. For instance, higher VSTs are preferred in environments with higher operational temperature conditions, while lower VSTs could be selected for applications for lesser thermally demanding applications [62].

**Figure 9:**
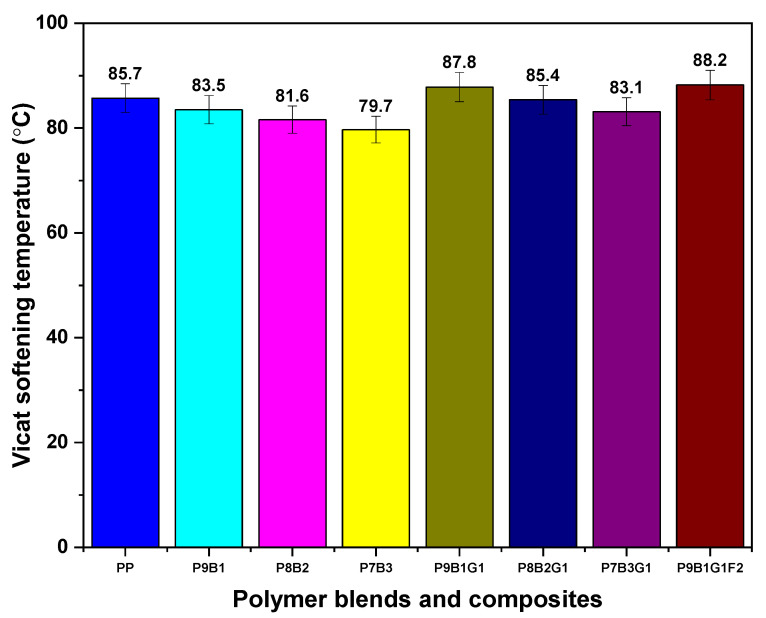
Vicat softening temperature of PP, PB, polymer blends, and their composites.

Figure 9 shows the VSTs of the polymers, polymer blends, and their composites. The results reported that increasing PB loading with PP (P9B1, P8B2, and P7B3) decreased VST values. These reductions in VSTs can be attributed to PB’s amorphous nature and elastomeric properties, which reduced the samples’ rigidity. However, incorporating GF and FF with polymer-blended samples improved the composites’ resistance and resulted in higher VST values, as reported in Figure 9. These improvements were attributed to the introduction of FF, which can offer additional benefits such as improved interfacial bonding that contribute to enhanced thermal stability [56]. For instance, the P9B1G1F2 composite sample reported the highest VST value of 88.2 °C among the tested samples.

## 4. Conclusions

This work proposed fabrication using PP, PB, GF, and FF. Fabrication was carried out using a twin-screw extruder followed by compression moulding techniques. The samples were fabricated by varying the polymer loading between the PP and PB. Additionally, GF and FF were reinforced with polymer blends to improve the mechanical and thermal performances of the composite samples. The significant findings from this study are reported below.

The PP sample exhibited higher tensile properties than the pure PB sample. In polymer blending, when the PB loading in PP was increased, the tensile properties of the polymer-blended samples were observed to decrease. For instance, the tensile strength ranged from ~12 MPa to ~18 MPa. The tensile modulus ranged from ~658 MPa to ~837 MPa. However, higher tensile properties were observed in the hybrid composite sample (P9B1G1F2).

Regarding the flexural properties, the polymer-blended samples exhibited lesser flexural properties when compared to pure PP. When increasing the PB loading to PP samples, the flexural properties were observed to decrease due to PB’s amorphous nature. However, this behaviour was improved by incorporating GF with polymer blends. Improved flexural properties were reported in the hybrid sample. The flexural strength and modulus of the hybrid samples were ~29 MPa and 868 MPa, respectively.

In impact strength, the polymer-blended samples were reported to have higher values than pure PP. The ranges of impact strength values in polymer-blended samples were 13.1 kJ/m^2^ to 20.5 kJ/m^2^, whereby the pure PP exhibited an impact strength of 11.8 kJ/m^2^. The impact strength was further improved when GF was incorporated with polymer-blended samples. A higher impact strength of 35.3 kJ/m^2^ was observed in the P9B1G1F2 hybrid sample.

From the DSC results, the PP exhibited a melting transition around 160 °C to 170 °C. The addition of PP and PB resulted in a lower and broader temperature melting curve being observed. This observation indicated a reduction in crystallinity due to PB’s amorphous nature. Further, adding PB to PP samples disrupted the PP’s crystallinity. When GF was introduced with polymer-blended samples, a slight increase in crystallinity was observed compared to pure PP. An enhancement in the crystallinity was noted by introducing FF with the relevant polymer blend and GF.

From the HDT and Vicat softening temperature plots, the P9B1G1F2 hybrid samples outperformed all other tested samples.

In future work, other natural fibres such as jute, hemp, and coir could be explored to enhance the sustainability of hybrid composites. Additionally, the impact of long-term ageing effects will be investigated to assess the durability of the samples. Improvements in fibre-to-matrix bonding through fibre surface treatments or the use of compatibilizers, along with rheological studies, will also be considered for future research.

## Figures and Tables

**Table 1:** Properties of PP, PB, GF, and FF.

Polymer/Fibre	Density (g/cm^3^)	Melting Temperature (°C)	Melt Flow Rate (g/10 min)	Tensile Strength (MPa)	Tensile Modulus (GPa)	Elongation at Break (%)
PP	0.89	160–170	3.0	20–25	1.3–1.8	100–600
PB	1.57	-	-	5–10	0.01–0.1	200–700
GF	2.57	600–800	-	2000–2500	70–85	2–3
FF	1.50	-	-	350–800	19.8	2.9

**Table 2:** Composition details of polymer blends and their composite samples for weight fraction.

Sample Code	PP Weight (%)	PB Weight (%)	GF Weight (%)	FF Weight (%)
PP	100	0	0	0
PB	0	100	0	0
P9B1	90	10	0	0
P8B2	80	20	0	0
P7B3	70	30	0	0
P9B1G1	90	10	10	0
P8B2G1	80	20	10	0
P7B3G1	70	30	10	0
P9B1G1F2	90	10	10	20

## Data Availability

The data presented in this study are available on request from the corresponding author due to privacy.
